# SLPI knockdown induced pancreatic ductal adenocarcinoma cells proliferation and invasion

**DOI:** 10.1186/s12935-015-0182-4

**Published:** 2015-04-01

**Authors:** Wei Zhang, Jian-Long Yao, Shan-Chao Dong, Feng-Qiang Hou, He-Ping Shi

**Affiliations:** Department of general surgery, The Weinan central hospital, Weinan, 714000 Shaanxi Province China

**Keywords:** Pancreatic ductal adenocarcinoma, Secretory leukocyte protease inhibitor, Small interference RNA, Cell proliferation, Apoptosis, Migration, Invasion

## Abstract

**Background:**

Pancreatic ductal adenocarcinoma (PDAC) is an aggressive disease and still continues to have the worst prognosis of all gastrointestinal malignancies. Reports have demonstrated that secretory leukocyte protease inhibitor (SLPI) is overexpressed in various cancers and may be a potential therapeutic strategy for the treatment of different cancers. However, the possible role of SLPI in PDAC is still unknown. In the present study, we investigate the effects of SLPI gene knockdown on the biological behavior of human pancreatic cancer cells. The expressions of SLPI were detected, by qRT-PCR and Western blot, in human PDAC tissues as well as AsPC-1, BxPC-3 and PANC-1 cells. After transfection with siRNA targeting to SLPI, SLPI expression was detected by qRT-PCR and Western blot in cells. Cell proliferation and apoptosis were also evaluated by MTT assay and flow cytometry (FCM). The trans-well assays were also employed to explore the effects of SLPI knockdown on the migration and invasion of PDAC cells *in vitro*.

**Results:**

The expressions of SLPI derived from human PDAC and PDAC cell lines were significant higher than those of control groups, respectively (*P* < 0.05). Regression analysis showed elevated SLPI level was positive correlated with development of PDAC. The siRNA target to SLPI significantly decreased the expressions of SLPI in these PDAC cell lines. Following SLPI-siRNA transduction, the proliferative capacity of the AsPC-1, BxPC-3 and PANC-1 cells was significantly inhibitions, compared to the blank (PDAC-wild type cells) and negative (non-targeting scrambled siRNA transduced PDAC cells) control ones, respectively (*P* < 0.05). Moreover, SLPI knockdown significantly increased the apoptosis fractions and reduced the migration and invasion of PDAC cells *in vitro* (*P* < 0.05).

**Conclusions:**

The present study demonstrated that: i) SLPI played an important role in PDAC progression; ii) SLPI might be an important characteristic of malignant PDAC associated with migration and invasion *in vitro*; and iii) siRNA targeting to SLPI might be a potential therapeutic strategy for the treatment of PCC.

## Background

Pancreatic ductal adenocarcinoma (PDAC) accounts for 95% of pancreatic cancer (PCC) and has a dismal prognosis, with only a 6% 5-year survival rate [1]. Despite considerable advances in radiological techniques, it often presents as a locally advanced or metastatic disease in most patients and only about 10-20% of patients are considered candidate to surgery [2]. Unfortunately, most of the patients with PDAC are diagnosed at an advanced stage due to the lack of obvious symptoms, and their prognosis remains very dismal [3,4]. Thus, early detection and diagnosis of PDAC still present the best chance for successful treatments and improved outcomes.

Secretory leukocyte protease inhibitor (SLPI) is present in human mucus secretions and tissues and produced primarily in the epithelial cells lining the respiratory, digestive and reproductive tracts [5,6]. SLPI, 11.7 kDa serine protease inhibitor, is belongs to the whey acidic protein four-disulfide core family of proteins. It binds heparin, a highly sulfated glycosaminoglycan also found in mast cell secretary granules, and the interaction increases its effectiveness as an inhibitor of neutrophil elastase [7]. SLPI was reported to be an anti-inflammatory factor that contributes at the early inflammatory response in odontoblasts [8]. In addition, SLPI was reported to play a role not only in protecting the tissues from the protease, but also in cell proliferation, inhibiting HIV infections, anti-bacterial and anti-fungal activity [9-11]. Gene-targeting experiments in mice indicated that one function of SLPI is to protect proepithelin from elastase cleavage in wound healing. Recent study showed that SLPI expression in relation to cancer progression, metastasis and invasion [5,12]. SLPI is also produced in cancer tissues and upregulated under tumorigenic conditions. It is often elevated in various cancer tissues including lung, head/neck and ovarian cancers [6]. However, its role in cancer is not well understood, especially in PDAC.

The present study aimed to investigate the impact of SLPI gene knockdown on the biological behavior of human PDAC cells. The expressions of SLPI were detected, by quantity real-time PCR (qRT-PCR) and Western blot, in human PDAC tissues, adjacent normal tissues and human PDAC AsPC-1, BxPC-3 and PANC-1 cell lines. Then, an expressional knockdown vector carrying a small interference RNA (siRNA) targeting the SLPI gene was constructed and transfected into these cells. After transfection, SLPI expressions were detected by qRT-PCR and Western blot. The cell proliferation and apoptosis fractions were also evaluated by MTT assay and flow cytometry (FCM). The trans-well assays were also employed to explore the effects of SLPI knockdown on the migration and invasion of PDAC cells *in vitro*.

## Results

### SLPI expression in PDAC and normal tissues

The patients ranged in age from 47 to 69 years, with a median age of 56 years. SLPI expression was lowest among health normal tissue and significantly increased in PDAC patients (chi^2^=14.73, *P* < 0.05). Those patients determined higher levels of SLPI in PDAC samples increased the risk of high pathological type (*P* < 0.05).

### SLPI elevated in human PDAC tissues and cells

**Figure 1:**
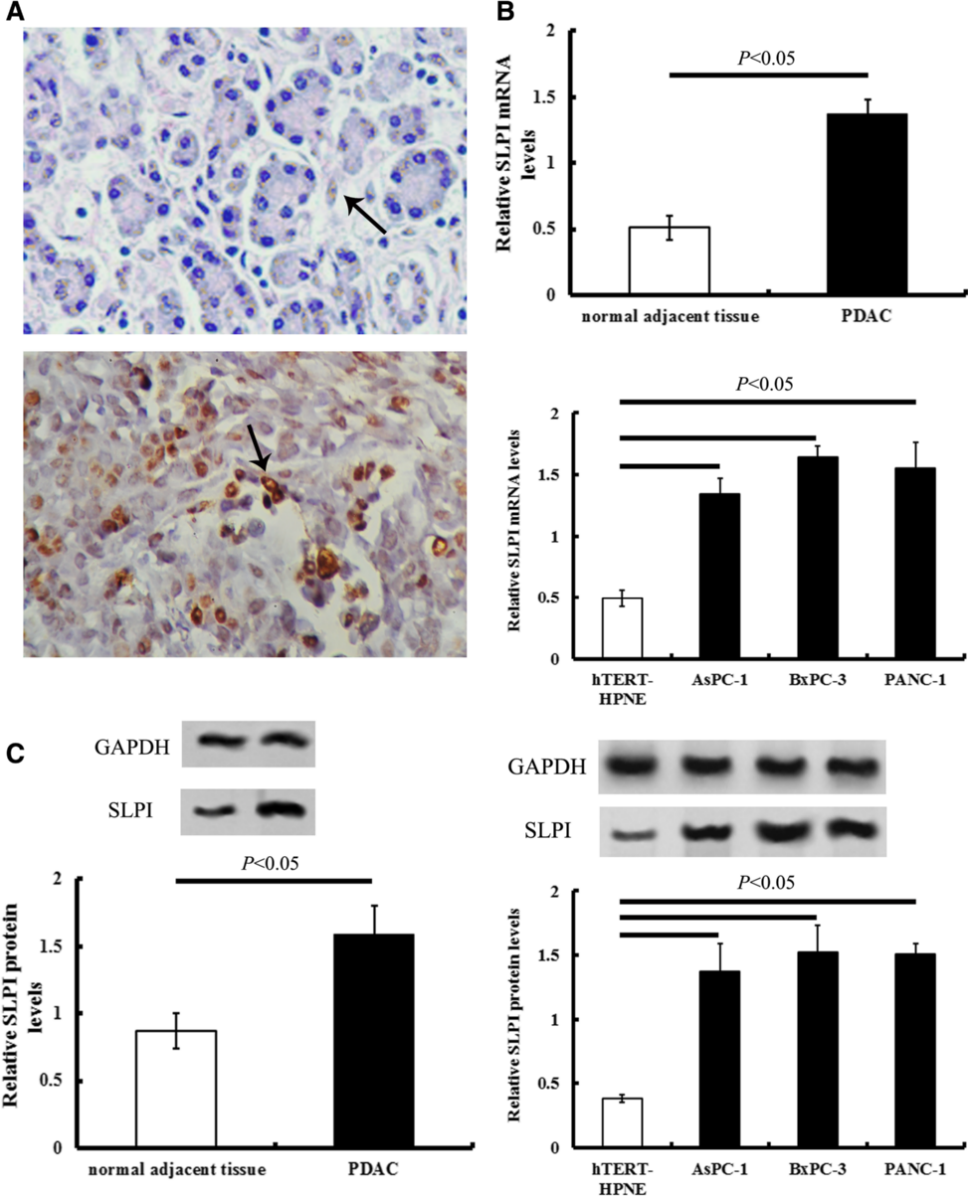
**IHC positive staining of SLPI in PDAC tissues and cell lines.** PDAC, pancreatic ductal adenocarcinoma tissue. **A**, Representative IHC files of SLPI in normal adjacent (up) and PDAC tissue (down) There is more intense yellow or yellow-brown staining for SLPI in PDAC tissue than that in normal adjacent tissue. **B**, the quantities analysis of relative mRNA levels of SLPI in PDAC tissue and cells. **C**, the representative immunoblotting photograph and the quantities analysis of protein levels of SLP. The arrow shows the representative immunostaining of SLPI. Magnification: x 200. Values plotted are means ± SD (n=45).

SLPI staining in normal adjacent tissue was weak relative to PDAC tissues. The IHC positive files of SLPI exhibited light yellow to brown staining (Figure 1A). Either qRT-PCR or Western blot analysis showed that the expressions of SLPI in PDAC tissue were significantly intensive than that of normal adjacent tissues (*P* < 0.05, Figure 1B and C).

Compared with that of normal human pancreas hTERT-HPNE cells, the mRNA and protein levels of SLPI in AsPC-1, BxPC-3 and PANC-1 increased significantly (*P* < 0.05, Figure 1B and C).

### Special SLPI target siRNA transduction induced inhibitions of SLPI

**Figure 2:**
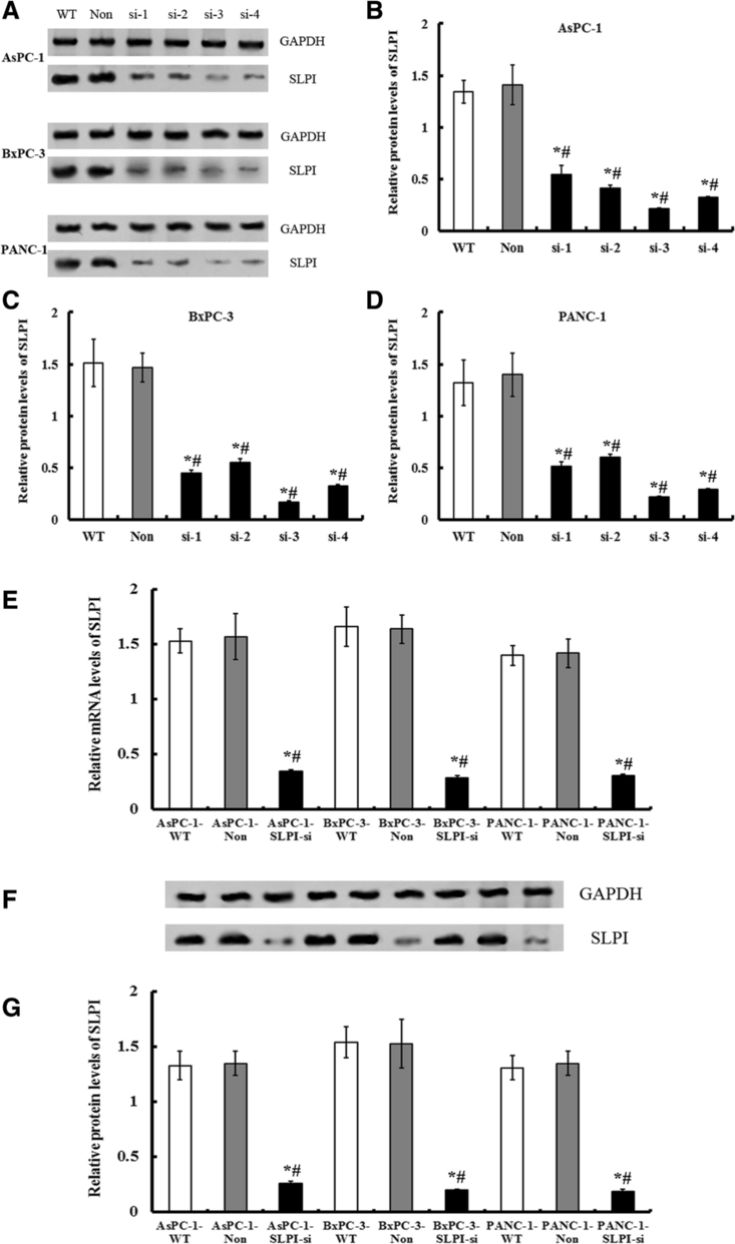
**Expressions of SLPI in various cell lines after SLPI-siRNA transduction. A**, the protein expressions of SLPI were evaluated by Western blot following SLPI knockdown by siRNA transfection. **B**, **C** and **D**, the quantities analysis of relative protein levels of SLPI after SLPI-siRNA treatment in three PDAC cell lines. GAPDH was used as the control. Considering the highest expressional inhibition rates in SLPI, si-3 was chosen as the target siRNA for followed investigation. **E**, relative SLPI mRNA levels detected by qRT-PCR were significantly lower in SLPI-siRNA expressed AsPC-1, BxPC-3 and PANC-1cells than the matched control (PDAC-WT or PDAC-Non), respectively (*P* < 0.05). **F**, the immunoblotting analysis of SLPI following SLPI knockdown. **G**, the quantities analysis of relative protein levels of SLPI after SLPI-siRNA treatment in three PDAC cell lines. Values plotted are means ± SD (n=3). * vs PDAC-WT cell lines, *P* < 0.05. # vs PDAC-Non cell lines, *P* < 0.05.

Stable expression of three SLPI siRNA (si-1, si-2, si-3 and si-4) in PDAC cells resulted in >75% decrease in SLPI expression (Figure 2A-D). Considering the highest expressional inhibition rates in SLPI, si-3 was chosen as the target siRNA for followed investigation.

The PDAC cell lines, including AsPC-1, BxPC-3 and PANC-1 cells, then were stably transfected with SLPI si-3 (named as AsPC-1-SLPI-si, BxPC-3-SLPI-si and PANC-1-SLPI-si, respectively). Negative control AsPC-1, BxPC-3 and PANC-1 cells were transfected with non-targeting siRNA vectors. They were recorded as AsPC-1-Non, BxPC-3-Non and PANC-1-Non, respectively. The wild type PDAC cells were also employed as the blank control, which was named as AsPC-1-WT, BxPC-3-WT and PANC-1-WT, respectively. SLPI mRNA levels detected by qRT-PCR were significantly lower in SLPI-siRNA expressed cells than the matched control ones (*P* < 0.05, Figure 2E). Western blot analysis found that the level of immunoreactive protein was significantly down-regulated in SLPI-siRNA transfected cells relative to the controls cells (*P* < 0.05, Figure 2G).

### Effects of SLPI-siRNA on PDAC cells proliferation

**Figure 3:**
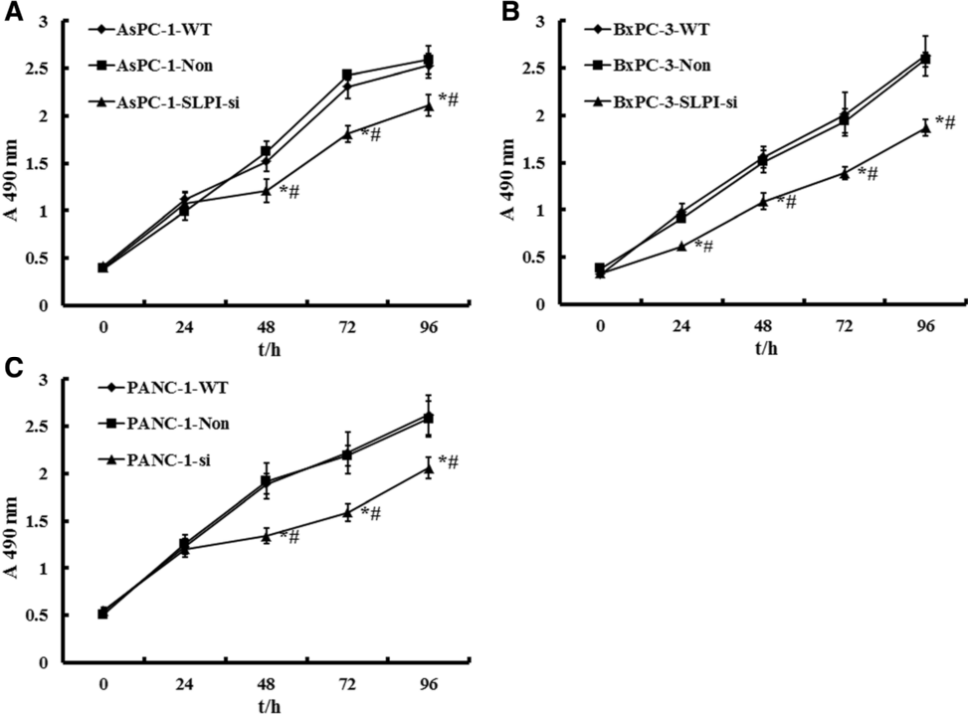
**Effects of SLPI on PDAC cell proliferation.** MTT assay time-course for WT and SLPI-silence AsPC-1 cells **(A)**, BxPC-3 **(B)** and PANC-1 cells **(C)**. After SLPI down-regulation, AsPC-1 cells and PANC-1cells showed significant decrease in proliferation compared with that of control ones (negative and blank control), respectively (*P* < 0.05). Values plotted are means ± SD (n=3). * vs PDAC-WT cell lines, *P* < 0.05. # vs PDAC-Non cell lines, *P* < 0.05.

We assessed the effect of SLPI expressional silence on the regulation of PDAC cells viability. MTT assay showed that SLPI expressional knockdown caused significantly decrease in cell viability in AsPC-1, BxPC-3 and PANC-1 cells, compared with that of the control ones, respectively (*P* < 0.05, Figure 3A, B and C).

### SLPI knockdown induced increase apoptosis of PDAC cells

**Figure 4:**
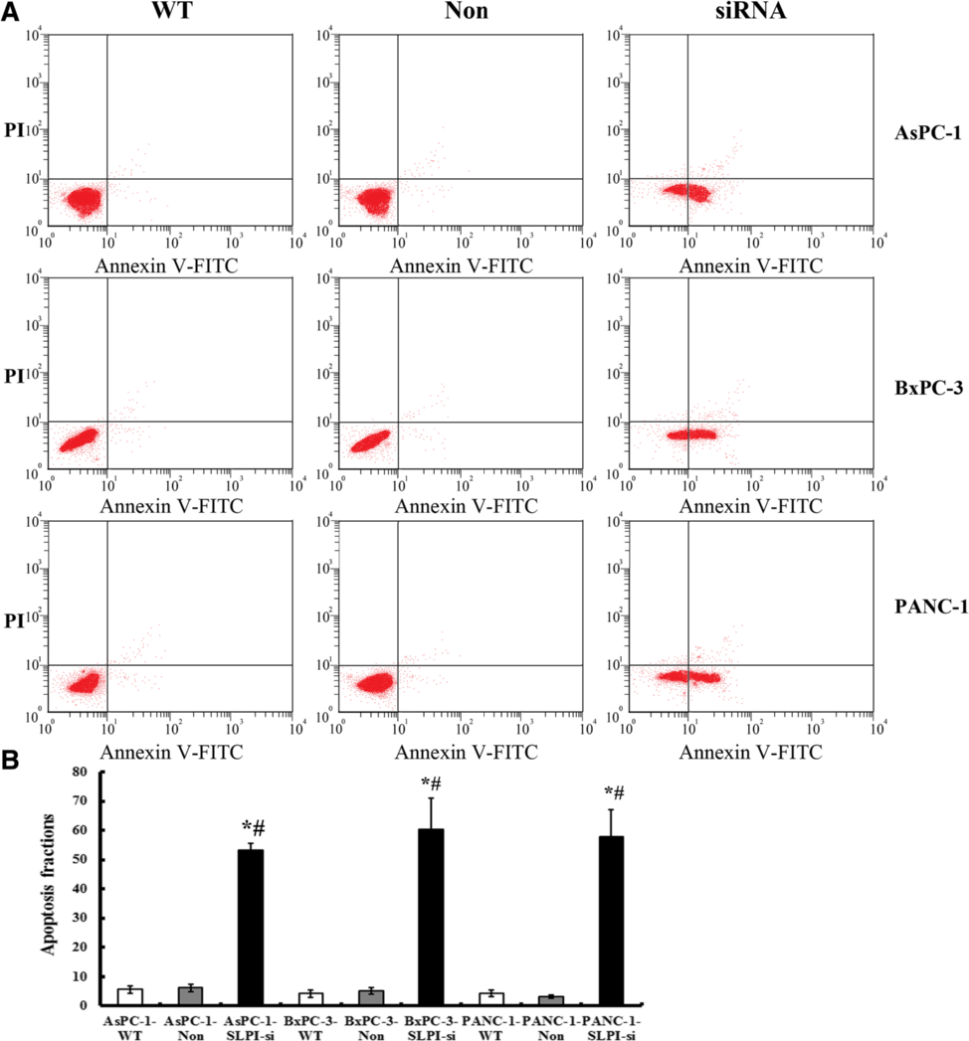
**SLPI knockdown induced apoptosis in PCC cells. A**, the influences of SLPI knockdown on the apoptosis of AsPC-1 cells, BxPC-3 and PANC-1 were detected by FCM, respectively. **B**, quantification shows that the percentage of apoptotic cells in SLPI-siRNA transfected group is significantly higher compared to that in the control groups. Values plotted are means ± SD (n=3). * vs PDAC-WT cell lines, *P* < 0.05. # vs PDAC-Non cell lines, *P* < 0.05.

There was a significant increase in the apoptosis rate in SLPI-siRNA infected cells relative to Empty infected ones detected by FCM (Figure 4). There were more apoptosis PDAC cells in AsPC-1-SLPI-siRNA, BxPC-3-SLPI-siRNA and PANC-1-SLPI-siRNA groups, when compared with that of the matched WT and non-targeting siRNA control groups, respectively (*P* < 0.05, Figure 4A and B).

### Effect of SLPI knockdown on PDAC cell migration

**Figure 5:**
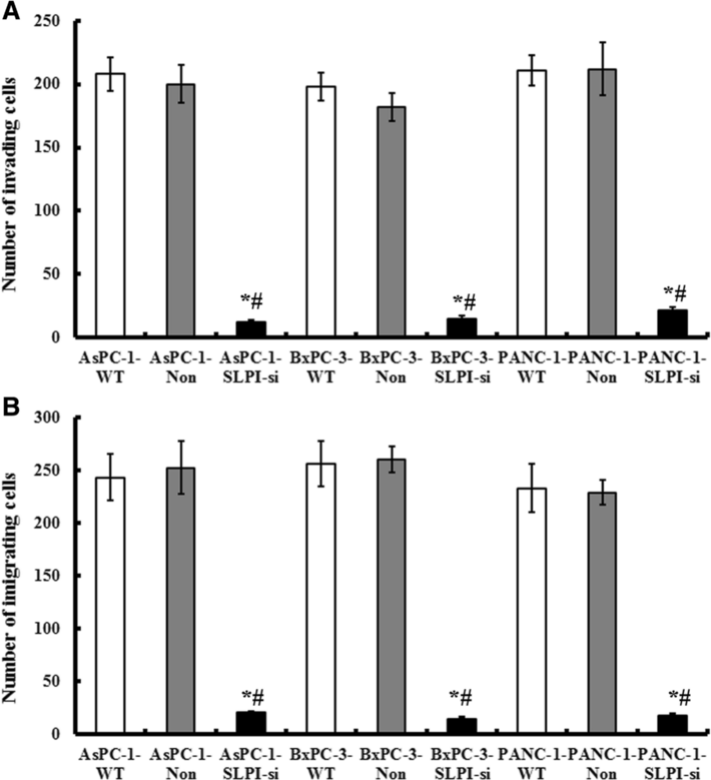
**The effects of SLPI knockdown on the migration and invasion of PDAC cell lines. A**, migration was expressed as number of migrating cells per 40X field. **B**, Invasion was expressed as number of invading cells per 40X field. Values plotted are means ± SD (n=3). * vs PDAC-WT cell lines, *P* < 0.05. # vs PDAC-Non cell lines, *P* < 0.05.

Following knockdown, we compared the migration of control non-transduced innocent cells (blank), non-targeting scrambled siRNA transduced cells (negative control), as well as the SLPI knockdown cells transduced with SLPI targeting siRNA. The migration assay showed that the crystal violet stained cells significantly decreased in the SLPI-siRNA treated cells, compared with that of the matched WT and non-targeting siRNA control groups (*P* < 0.01, Figure 5A). The SLPI knockdown treatment significantly decreased the migration of the three cell types compared to the negative and blank control ones. Through the whole experimental duration, migration was not significantly different between AsPC-1, BxPC-3 and PANC-1 blank cells and the negative control cells transduced with non targeting scrambled siRNA, as shown in Figure 5A.

### Effect of SLPI knockdown on PDAC cell invasion

There were significant reductions in the invasion of AsPC-1, BxPC-3 and PANC-1 cells following SLPI knockdown, in comparison with that of the control cells, respectively (*P* < 0.005) (Figure 5B). The invasion of control cells transduced with non-targeting siRNA, which had unchanged levels of SLPI, was not significantly different from the non-transduced WT PDAC cells.

## Discussion

The current study showed that SLPI elevated in human PDAC tissue and cell lines, including AsPC-1, BxPC-3 and PANC-1 cells. Increased expression of SLPI was positively correlated with the PDAC development. Target SLPI-siRNA transduction in AsPC-1, BxPC-3 and PANC-1 cells induced: i) significant inhibitions in proliferation; ii) increase apoptosis fractions; and iii) the reductions of migration and invasion of PDAC cells. These data suggested that SLPI might play an important role in PDAC progression, and siRNA targeting of SLPI might be a potential therapeutic strategy for the treatment of PDAC.

It is well established that SLPI protects tissue from proteases, and promotes cell proliferation and healing during inflammatory response. In replicated proteomic datasets, the SLPI protein stood out based on its decrease in abundance in both oral premalignant lesion tissue (OPMLs) and oral squamous cell carcinoma (OSCC) lesion tissues compared to healthy normal tissue [13]. By multiplex proximity ligation assay, Simon Fredriksson et al. had demonstrated that SLPI was overexpressed in plasma samples from PCC compared than the age-matched controls, and in behaved as high-abundance markers for pancreatic cancer [14]. In the present, we found that the mRNA and protein levels of SLPI were significantly unregulated in human PDAC tissue and PDAC cell lines, AsPC-1, BxPC-3 and PANC-1 cells. Following target SLPI-siRNA transduction, cell proliferation of AsPC-1, BxPC-3 and PANC-1 cells was significantly inhibited, which was coincident with the increase apoptosis fractions. These facts suggested that SLPI might play a critical role in the progression of tumor growth and proliferation in human PDAC.

Moreover, our data also revealed that SLPI knockdown lead significant reduction in migration and invasion of PDAC cells, which might indicate an import role of SLPI involved in metastasis of PDAC. Recent reports revealed that SLPI is also overexpressed in gastric, lung and ovarian cancers, which accelerates the metastasis of cancer cells. The overexpression of SLPI was involved in metastasis of lung carcinoma 3LL-S cells [15]. Other researches showed that SLPI, expressed in a range of cancer cell lines, was particularly overexpressed in the highly liver metastatic tumor [16]. It is also revealed SLPI is overexpressed in rarely developed inflammatory breast cancer with highly angiogenic and metastatic capacity [17]. Additionally, the overexpression of SLPI in lung carcinoma cells showed rapid tumorigenicity and lung metastasis upon subcutaneous inoculation compared to the control cells [15]. Moreover, the blood concentration of SLPI was high in ovarian cancer compared to normal ovaries or benign ovarian tumors, and is particularly high in malignant tumors with a high metastatic potential [18]. It also demonstrated that SLPI mRNA was overexpressed in serosa invading gastric cancer cells, and the cell migration and invasion rate was significantly increased in a SLPI overexpressing gastric cancer cell line [19]. Regarding these, it is reasonable to postulate that SLPI might be associated with the metastatic potential of malignant PDAC and a potential therapeutic target. However, the mechanism underlying still needed further investigations.

## Conclusions

In conclusion, the present results showed that SLPI was overexpressed in human PDAC tissues and PDAC cell lines. SLPI knockdown by target siRNA introduction induced significantly inhibitions in proliferative capacity, increase in apoptosis, and reductions of migration and invasion of AsPC-1, BxPC-3 and PANC-1 cells. These findings suggested that: i) SLPI played an important role in PDAC progression; ii) SLPI might be an important characteristic of malignant PDAC associated with migration and invasion *in vitro*; and iii) siRNA targeting to SLPI might be a potential therapeutic strategy for the treatment of PCC.

## Materials and methods

### Clinical samples collections

Through the surgery consent form, patients were informed that the resected specimens were kept by our hospital and might be used for scientific research, and that their privacy would be maintained.

Forty-five patients attending the clinic in the central Hospital of Weinan City in Shanxi Province, between February/2013 to May/2014, were invited to participate in the study. The patients ranged in age from 46 to 75 years, with a median age of 54 years. All of them accepted the operation for the first time, without any anti-tumor treatment. The specimens were histopathologically verified as PDAC by senior pathologists. Then the tumor samples and matched normal adjacent tissues were obtained, which were taken at least 0.5 cm distal to tumor margins. The biopsies obtained were divided into two fragments immediately after surgery. One fragment was immediately stored at −80°C until nucleic acids and proteins isolation. The remaining fragment was fixed in 4% formaldehyde for two days and then was paraffin-embedded. The paraffin blocks were sliced and stained with immunohistochemistry (IHC) for histological examination.

### IHC staining

Immunohistochemistry was performed to determine the SLPI expression in normal adjacent tissue and PCC tissues, as described followed. Briefly, tumor samples and the matched control tissues were fixed in 4% formaldehyde, embedded in paraffin wax, and then cut into 4 μm sections using a microtome. These frozen sections were incubated with 2% goat serum at 37°C for 20 min, followed by incubation with goat-anti-human SLPI (1:500, Santa cruz, sc-10535) at 4°C overnight. After washing with PBS, the sections were incubated with horseradish peroxidase (HRP)-conjugated rabbit anti-rat IgG (HRP-IgG) at 37°C for 30 min and colored with 3, 3′-Diaminobenzidine (DAB) at room temperature. PBS was substituted for the anti-SLPI antibody in negative control subjects.

### Cell culture

Human PDAC cell lines AsPC-1, BxPC-3 and PANC-1, as well as normal human pancreatic hTERT-HPNE cells were purchased from Cell Bank of the Chinese Academy of Sciences. All of these cells were cultured in specific medium supplemented with 10% (v/v) fetal bovine serum (FBS) and 1% antibiotics at 37°C in a humidified incubator under 5% CO_2_ condition.

### Quantitative reverse-transcription PCR

The expressions of SLPI in the PCC specimens and the cell lines were detected by Quantitative real-time PCR (qRT-PCR). Tumor samples (50 mg) were ground under liquid nitrogen, lysed with 1 ml of Trizol (Takara, Japan), and total RNA was extracted using Trizol (Invitrogen, USA). Total RNA (2 μg) was added to the tumor extract with Moloney Murine Leukemia Virus Reverse Transcriptase (MMLV-RT, Takara, Japan) to synthesize cDNA, and the reverse transcript was used as the template for qRT-PCR using a Tower qRT-PCR system (Analytic Jena, Germany). The qRT-PCR was conducted using 2 × Mix SYBR Green I (Biosea, USA) (10 μl), primer (0.25 μl, 10 pmol/L), template DNA (1 μl), and sterile water (8.5 μl). All PCR reactions included initial denaturation and multiple cycles at (95°C for 3 min); 39 cycles at 95°C for 10 s, 54°C for 10 s, and 72°C for 30 s; followed by 95°C for 10 s, 65°C for 5 s, and a final 95°C for 15 s. The primer for each gene was synthesized by Invitrogen (USA). The real time PCR primers used to quantify GAPDH expression were: F*:* 5′-CGAGATCCCTCCAAAATCAA-3′ and R: 5′-TTCACACCCATGAC- GAACAT-3′ and for SLPI were: F 5′-CCCTTCCTGGTGCTGCTT-3′ and R: 5′- CCTCCTTGTTGGGTTTGG-3′. Expression of SLPI was normalized to endogenous GAPDH expression.

### Western blot

SLPI protein levels both in PDAC tissues and cell lines were determined by Western blot. Briefly, samples were lysed for 30 min in CytoBuster Protein Extraction Buffer (Novagen, USA) and centrifuged at 12000 rpm. The supernatant was collected, total protein was measured, and 50 μg was used for 10% sodium dodecyl sulfate polyacrylamide gel electrophoresis (SDS-PAGE). The protein was then transferred to a nitrocellulose (NC) membrane and sealed with Tris-Buffered Saline Tween-20 (TBST) containing 5% non-fat milk powder. The membrane was subsequently incubated with goat anti-human SLPI proteins and mouse anti-human GAPDH (1:500, Santa cruz, sc-81545) at 4°C overnight. After washing in TBST, the membrane was incubated with HPR conjugated secondary antibodies (1:2000) at 25°C, and the protein quantity was determined using electrochemiluminescence (ECL) technique (BestBio, USA). The results were photographed using the JS Gel Imaging System (Peiqing, China) and the grey density was calculated using SensiAnsys software (Peiqing, China).

### SLPI gene knockdown

According to the CDS of SLPI recorded in Neuclpeptide, we predesigned small interference RNA (siRNA) targeting the human SLPI gene (Gene ID, 10103) (http://RNAiDesigner.invitrogen.com). The siRNA sequences targeting SLPI are as follows. si-1: 5′-AAGCTGGAGTCTGTCCTCCTAAGAA-3′, si-2: 5′-CAGTGCA- AGCGTGACTTGAAGTGTT-3′, si-3: 5′-TCAAAGCTGGAGTCTGTCCTCCTAA-3′, si-4: 5′-CAAAGCTGGAGTCTGTCCTCCTAAG-3′. A scrambled non-target siRNA was also used as a control. Lentivirus was packaging in 293 T cells using Lipofectamine2000 (Invitrogen, Carlsbad, CA) and virus titers were determined. The interference efficiency of si-1-4 targeting SLPI in 293 T cells was determined by qRT-PCR and Western blot. The target siRNA was chosen for further investigation as it had the highest interference efficiency. Then the cell lines including AsPC-1, BxPC-3 and PANC-1 cells, were then infected with 1 × 10^6^ recombinant lentivirus-transducing units containing the target siRNA or non-targeting siRNA in the presence of 6 μg/ml polybrene (Sigma), respectively.

### Tetrazolium salt 3-(4,5-dimethylthiazol-2-yl)-2,5-diphenyltetrazolium bromide (MTT) assay

Cell viability was determined using the MTT assay. Briefly, cells were plated into 96-well culture plates at an optimal density of 5 × 10^3^ cells/mL in 200 μL of culture medium per well. After 24–96 h of culture, 20 μL of 5 mg/mL MTT was added to each well and incubated at 37°C for 4 h. The medium was then gently aspirated and 150 μL of dimethyl sulfoxide (DMSO) was added to each well to solubilize the formazan crystals. The optical density of each sample was immediately measured using a microplate reader (BioRad, Hercules, CA, United States) at 490 nm.

### Apoptosis assay

A propidium iodide (PI) and annexin V-FITC-flow cytometry assay (BD Pharmingen) was used to detect the apoptosis rate in the cells after SLPI transfection. Briefly, 1 × 10^6^ cells per well were cultured in 6-well plates in the absence of 10% FBS for 48 hours. Adherent cells were detached with 0.25% trypsin without EDTA in 1 × PBS. Cells were harvested in complete RPMI 1640 medium and centrifuged at 1000 rpm for 5 minutes. Each of the cells were washed with 1 × PBS and stained with 50 ug/ml PI and Annexin V-FITC, following the manufacturer’s instructions.

### Cell migration and invasion assay

Biocoat matrigel invasion chambers (BD Biosciences, Bedford, MA, United States) were used to compare the effect of SLPI knockdown on *in vitro* invasion of AsPC-1, BxPC-3 and PANC-1 cells as previously described [20,21]. Briefly, for the invasion assay, Costar Tran-swell 8 μm inserts were coated with 50 μg reduced serum Matrigel (BD Biosciences, Bedford, MA, United States). Invasion Chambers (BD China, Shanghai, China) were coated with Matrigel, and 1 × 10^6^ cells were added per chamber. Medium supplemented with 10% FBS was used in the lower chamber. Following incubation cells that had invaded through the membrane were fixed and stained before the membrane was removed and mounted on a slide for microscopic assessment. Invasive cells were visualized at x40 magnification and the number of cells in five random fields was counted and an average calculated.

For migration assays, the same procedure was used excluding the Matrigel. After 12 h, non-invading cells and media were removed, and cells on the lower surface of the membrane were fixed with polyoxymethylene (Sigma) and stained with 0.1% crystal violet (Sigma) for 0.5 h. Stained cells were counted under a microscope in five randomly selected fields, and the average was used to indicate cell migration and invasion. All experiments were performed in triplicate [21].

### Statistical analysis

SPSS v11.5 (SPSS Inc., Chicago, IL) was used for statistical analysis. Data are presented as means ± standard deviation. Statistical significance was determined by the t-test, One-way ANOVA analysis, followed by the Fisher Halves test for other data analyses. A *P* < 0.05 was considered statistically significant.
